# CHILDHOOD SEXUAL TRAUMA, SUBSTANCE USE, AND ART ADHERENCE AMONG OLDER ADULTS LIVING WITH HIV

**DOI:** 10.1093/geroni/igac059.1042

**Published:** 2022-12-20

**Authors:** Monique Brown

**Affiliations:** University of South Carolina, Columbia, South Carolina, United States

## Abstract

Childhood sexual abuse (CSA) is associated with lower ART adherence among people living with HIV and is linked to substance use (SU) in adulthood. However, studies examining the mediating role of SU in the association between CSA and ART adherence are lacking. Therefore, the aim of this study was to examine SU as a mediator in the association between CSA and ART adherence among older adults living with HIV (OALH). Data were obtained from 91 OALH. Adjusted path models were used to determine the mediating role of SU. After controlling for sociodemographic characteristics and depression, CSA had a direct association with ART adherence (β=-3.20; p<0.001) and with SU (β=-2.46; p=0.023). The association between SU and ART adherence was not statistically significant (β=0.071; p=0.444). Therefore, the indirect effect between CSA, SU, and ART adherence was not statistically significant (β=0.175; p=0.468). Future research should examine alternative pathways between CSA and ART adherence.

